# Superficial siderosis and the dura

**DOI:** 10.1111/ene.16182

**Published:** 2023-12-14

**Authors:** Wouter I. Schievink

**Affiliations:** ^1^ Department of Neurosurgery Cedars‐Sinai Medical Center Los Angeles California USA

Derived from the Greek word *σíδηρος* (sideros) meaning “iron”, superficial siderosis (SS) is characterized by hemosiderin deposits in the leptomeninges and subpial layers of the brain, cranial nerves, and spinal cord [1]. SS has always been a perplexing disorder for neurologists and neurosurgeons alike, but the 2017 landmark study by Wilson et al. brought much needed clarity [2]. Those authors were able to untangle the clinical and radiographic characteristics of SS and distinguished the classic type of SS, also known as primary infratentorial SS (iSS), from supratentorial SS that is caused by cerebral amyloid angiopathy. Primary iSS is centered in the superior cerebellum and is not associated with a history of intracranial hemorrhage. It is this iSS type that is associated with the classic triad of progressive hearing loss, ataxia, and myelopathy, although other symptoms may be present as well, such as cognitive impairment, tinnitus, and anosmia. The cause of iSS is chronic bleeding within the subarachnoid space.

Largely through the work of Kumar, it has become well established that dural and not vascular pathology is by far the most common cause of iSS and that it is more often at the level of the spine than at the level of the cranium [3]. The underlying dural pathology can be divided into two main categories: (i) the spontaneous type, for example, a spontaneous spinal cerebrospinal fluid (CSF) leak, oftentimes with a remote history of orthostatic headaches typical of spontaneous intracranial hypotension (SIH) or dural ectasia associated with connective tissue disorders such as Marfan syndrome or ankylosing spondylitis, and (ii) the traumatic type, for example, a CSF leak due to nerve root avulsion associated with brachial plexus injury or an iatrogenic CSF leak usually in the form of a pseudomeningocele following spinal surgery.

In this issue of the *European Journal of Neurology*, Rahal et al. provide further evidence for the interrelationship of spontaneous spinal CSF leaks and iSS and, moreover, show that repair of the spinal CSF leak can improve or at least arrest progression of iSS symptoms [4]. In their study of 142 patients with SIH, 95 had a ventral leak and 12 of those 95 (12.6%) had iSS, compared to none of the 47 patients with a different type of spontaneous CSF leak. These results are remarkably similar to those in our practice in which we found iSS in 46 of 447 patients (10.3%) with a ventral leak compared to less than 1% (11 out of 1142) of patients with a different type of spontaneous spinal CSF leak [5]. The median latency between onset of the CSF leak and demonstration of iSS also was very similar between these two groups of patients (9.5 vs. 10.5 years) [4, 5]. Then, how do we explain the apparent discrepancy between these two groups of patients in the reported improvement of iSS symptoms following surgical repair of the CSF leak (70% vs. 25%)? [4, 5] It is likely caused by the difficulty, even among experienced physicians, of discerning the underlying cause (iSS or SIH) of symptoms common to both disorders, particularly hearing loss and gait unsteadiness. In the study by Rahal et al., symptoms were attributed to iSS in 10 (83%) of 12 patients with iSS [4], while we attributed symptoms to iSS in 18 (39%) of 46 patients with iSS [5]. The truth likely lies somewhere in between.

Many uncertainties remain in the evaluation and treatment of patients with or at risk of iSS. One dilemma in the decision‐making process is what to do when confronted with a patient with iSS and no extradural CSF collection indicating a ventral CSF leak or other pathology, such as a spinal ependymoma on magnetic resonance imaging (MRI), that can explain the presence of the iSS. If other stigmata of SIH are present on brain MRI, such as meningeal enhancement, then the decision is straightforward and specialized imaging to search for a CSF‐venous fistula is warranted. These fistulas are a common cause of SIH, but are not associated with extradural CSF, and require specialized imaging such as digital subtraction myelography or dynamic CT myelography for their detection [5]. However, if MRI of the neuraxis is normal and there is no history of SIH, then specialized imaging searching for a CSF‐venous fistula as well as catheter craniospinal angiography should be considered to search for that rare vascular cause of iSS [6]. Idiopathic iSS does not exist.

Another dilemma is presented by the patient with a ventral spinal CSF leak who does not have iSS and has no or only minimal symptoms of SIH. The most reliable estimate of the likelihood of developing iSS in the setting of such a persistent ventral leak is approximately 50% over 15 years [7]. Epidural blood patches usually fail in the treatment of ventral CSF leaks unless performed early in the disease course, and surgery is the only option. Because the success rate of surgery to repair a ventral CSF leak in experienced hands is high and the complication rate of this surgery is low [5, 8], we typically recommend surgery to obviate the risk of developing iSS.

## AUTHOR CONTRIBUTIONS

**Wouter I. Schievink:** Conceptualization; writing – original draft; writing – review and editing.

## CONFLICT OF INTEREST STATEMENT

None.
